# *Leishmania major* Infection in Humanized Mice Induces Systemic Infection and Provokes a Nonprotective Human Immune Response

**DOI:** 10.1371/journal.pntd.0001741

**Published:** 2012-07-24

**Authors:** Anja Kathrin Wege, Christian Florian, Wolfgang Ernst, Nicole Zimara, Ulrike Schleicher, Frank Hanses, Maximilian Schmid, Uwe Ritter

**Affiliations:** 1 Institute of Immunology, University of Regensburg, Regensburg, Bavaria, Germany; 2 Clinic of Gynecology and Obstetrics St. Hedwig, University of Regensburg, Regensburg, Germany; 3 Microbiology Institute-Clinical Microbiology, Immunology and Hygiene, University Hospital of Erlangen and Friedrich-Alexander-University Erlangen-Nuremberg, Erlangen, Germany; 4 Department of Internal Medicine I, University Hospital Regensburg, Regensburg, Germany; Universidad Autónoma de Yucatán, Mexico

## Abstract

**Background:**

*Leishmania (L.)* species are the causative agent of leishmaniasis. Due to the lack of efficient vaccine candidates, drug therapies are the only option to deal with cutaneous leishmaniasis. Unfortunately, chemotherapeutic interventions show high toxicity in addition to an increased risk of dissemination of drug-resistant parasites. An appropriate laboratory animal based model is still missing which allows testing of new drug strategies in the context of human immune cells *in vivo*.

**Methodology/Principal Findings:**

Humanized mice were infected subcutaneously with stationary phase promastigote *L. major* into the footpad. The human immune response against the pathogen and the parasite host interactions were analyzed. In addition we proved the versatility of this new model to conduct drug research studies by the inclusion of orally given Miltefosine. We show that inflammatory human macrophages get infected with *Leishmania* parasites at the site of infection. Furthermore, a *Leishmania*-specific human-derived T cell response is initiated. However, the human immune system is not able to prevent systemic infection. Thus, we treated the mice with Miltefosine to reduce the parasitic load. Notably, this chemotherapy resulted in a reduction of the parasite load in distinct organs. Comparable to some Miltefosine treated patients, humanized mice developed severe side effects, which are not detectable in the classical murine model of experimental leishmaniasis.

**Conclusions/Significance:**

This study describes for the first time *L. major* infection in humanized mice, characterizes the disease development, the induction of human adaptive and innate immune response including cytokine production and the efficiency of Miltefosine treatment in these animals. In summary, humanized mice might be beneficial for future preclinical chemotherapeutic studies in systemic (visceral) leishmaniasis allowing the investigation of human immune response, side effects of the drug due to cytokine production of activated humane immune cells and the efficiency of the treatment to eliminate also not replicating (“hiding”) parasites.

## Author Summary

As many as 12 million people suffer from *Leishmania* (L.) infection worldwide with about one to two million newly infected people every year. Due to the lack of vaccine strategies, the only option is chemotherapeutic intervention which can cause serious side effects. Therefore, new prevention or treatment strategies are urgently needed in addition to an appropriate animal model for testing. We infected humanized mice in the footpad with stationary phase promastigote *L. major* and analyzed the human innate and adaptive immune response by flow cytometry, histology, and quantitative PCR. Infected macrophages were detectable at the site of infection and in lymphoid organs. Additionally, we were able to measure *Leishmania*-specific T cell priming in humanized mice. However, these human immune defense mechanisms were not sufficient to prevent systemic spreading and lethality. By the inclusion of Miltefosine, we tested this novel model for its versatility in conducting drug studies. The oral treatment was able to reduce parasitic load but also revealed side effects which are described in humans but not in mice. Therefore, we propose humanized mice as a novel model, which offers the opportunity to study new therapy strategies in chronic leishmaniasis in the context of a human immune system.

## Introduction

Over the last decades inbred mice were used to investigate the mechanisms of adaptive and innate immune responses against the obligatory intracellular parasite *Leishmania* (*L*.) *major*. From these studies fundamental paradigms regarding the elimination of the parasites were generated [1]. It could be demonstrated that in resistant C57BL/6 mice the elimination of intracellular parasites depends on the induction of an interferon-γ (IFN-γ) -driven T helper (Th) 1 immune responses. This Th1-associated cytokine production is crucial for the activation of infected macrophages to produce leishmanicidal pathways such as inducible nitric oxide synthase (iNOS) and subsequently nitric oxide radicals (NO) [2]. In contrast non-healing BALB/c mice mount a Th2 response associated with high levels of IL-4 and IL-13 [3]–[5]. Of note, similar to the experimental model, healing of cutaneous leishmaniasis in humans is also associated with a Th1-type immune response [5].

However, leucocytes from humans and rodents vary in their phenotype and function. For instance, the number of granulocytes in peripheral blood, which are the first line of defense against *Leishmania* infection, differs between human (50–70%) and mouse (10–25%) [6]. Furthermore, the relevance of the leishmanicidal molecules such as NO that is known to be substantially involved in the elimination of *L. major* parasites in the murine model of experimental leishmaniasis, is still not completely understood in the human system [7]–[11]. This might be due to the fact that murine macrophages respond to classical iNOS-inducing stimuli such as LPS and IFN-γ with iNOS expression and accumulation of NO whereas in humans alternative stimuli (such as IFN-α/β, IL-4, and anti-CD23) appear to be more efficient for monocyte and macrophage stimulation [2], [12]–[13]. In addition antimicrobial peptides e.g. defensins which have the potential to reduce intracellular pathogens [14] differ in number, sequence, genomic location, and activation between human and mice [2], [12]–[13]. For instance human defensins are present in human neutrophils but not in mice [15] whereas the mouse Paneth cells in the crypts of the small intestine express more than 20 different defensins but only two defensins are described in human cells [16].

Furthermore, differences in the maturation and regulation of T cells [17]–[18], in immunoglobulin subtypes, receptors, and their function [18] impede the transfer of scientific results gained from animal experiments to the human system.

NOD/scid-IL2Rγ^−/−^ (NOG or NSG) mice were first described and generated by crossing IL2Rγ^−/−^ mice with NOD-*scid* mice [19]–[20]. These mice lack not only B and T cells, but also feature considerably higher engraftment rates with human hematopoietic cells, extended lifespan, low levels of murine innate immunity and no NK cell activity [21] compared to previous mouse models. Upon transplantation of human CD34^+^ hematopoietic stem cells, NSG mice develop all human subsets of the immune system like T, B, and NK cells as well as myeloid cells and are able to mount an innate and adaptive immune response to antigens [19], [21], [22]. Therefore, mice bearing a human immune system might offer the opportunity to bridge the gap between animal models and clinical studies and are increasingly integrated in infectious disease studies and the investigation of human medication and vaccine projects [23]–[33].

The present study characterizes the human immune response in humanized mice after subcutaneous infection with *L. major* parasites. Our data reveal that human macrophages harbor *Leishmania* parasites. Unexpectedly, the parasitic replication was not limited to the site of infection but also observed in visceral organs such as spleen and liver. Although *Leishmania*-specific human T cells were generated, the humanized mice succumbed to the cutaneous infection.

To demonstrate the versatility of *Leishmania*-infected humanized mice for future drug studies, we complemented the infection studies with systemic Miltefosine treatment. Miltefosine efficiency was first implemented in tumor treatments and later described as a new line of drugs against *Leishmania* infections tested in humans [34] and mice [35]. It amends the second line drugs of aromatic diamidines, amphotericin B, and pentavalent antimonials which induce rising resistance and are toxic with severe sometimes life-threatening side effects [36].

In conclusion we could demonstrate that human immune cells interact with protozoan parasites in a murine environment. This novel experimental model might be beneficial for the investigation of drug efficiency to eliminate the parasite in the context of human immune cells possibly involved in severe side effects such as organ damage.

## Methods

### Ethic statement

Cord blood samples were taken with approval from the *Ethics* Committee of the University Regensburg (permission no. 08/021). All patients included in the experiments provided written informed consent. All experiments were performed in accordance with relevant institutional and national guidelines, regulations and approvals.

### Humanized mice

Mice (NOD-*scid IL2Rγ* ^null^; (NSG)) used for the experiments were obtained from Jackson Laboratories, and bred and kept in a specialized pathogen-free facility. Newborn mice were transplanted with 3×10^5^ CD34^+^ hematopoietic stem cells isolated from Cord blood as described before [37]. Hematopoietic cells were injected into the liver of neonatal mice. For infection studies, a total of 32 humanized mice, between three and five months of age were analyzed for their reconstitution levels shortly before *L. major* infection. All animal work was approved by the local veterinary authorities from the district government of Upper Palatinate/Bavaria based on the international European guidelines and national regulations of the German animal protection act (permission no. 54-2531.2-18/08).

### Infection and Miltefosine application

Humanized mice were infected via s.c. injection of 3×10^1^–3×10^6^ stationary phase promastigote *L. major* (MHOM/IL/81/FE/BNI) parasite into the right hind footpad adjusted to a final volume of 30 µl. BALB/c and C57BL/6 mice were infected with 3×10^6^ stationary phase promastigote *L. major* and served as controls. Disease progression was monitored on a daily basis. Footpad swelling and weight loss was monitored once a week. Treatment survey was implemented by administering Miltefosine (2.5 mg/kg; Cayman Chemical) orally on a daily basis using a gavage tube starting two weeks post infection. This initial condition of infection is important to mimic the scenario in *L. major* patients before starting Miltefosine treatment.

### Determination of parasite load using quantitative RT-PCR

Parasite load was determined using qPCR-analysis as described before [38]. SYBR Green (BioRAD, Germany) was used for product detection and quantified on total human-beta-actin 5′-GGG TGT AAC GCA ACT AAG TCA T-3′ (forward) and 5′-TGG ACA TCC GCA AAG ACC TG-3′ (reverse) or mouse ß-actin 5′-GGA TGC CAC AGG ATT CCA TAC CCA-3′ (reverse) and 5′-TCA CCC ACA CTG TGC CCA TCT ACG A-3′ (forward).

Amplification and detection were performed using the Multicolor Real-Time PCR Detection System (BioRAD, Germany). Standards, samples and non template controls were analyzed in triplicate for each run. As described in [38] the copies of *Leishmania* DNA (Units) were determined within the desired samples. DNA isolated from a distinct number of promastigote *Leishmania* parasites were used to determine the number of parasites/Unit *Leishmania* DNA. According to that internal standard we calculated the number of parasites within the sample. The data are presented as number of parasites/ng human β-actin (humanized mice) or number of parasites/ng mouse β-actin (C57BL/6 and BALB/c mice).

### Mononuclear cell isolation from various tissues

For FACS analyses mononuclear cells were isolated from the indicated tissues as previously described [37].

### T cell proliferation and activation assay

Spleen cells from infected humanized mice (six weeks post infection) and uninfected controls were isolated as described above and labeled with Carboxyfluorescein succinimidyl ester (Invitrogen, Oregon, USA) as described before [38]. For stimulation, 5×10^5^ cells/well from each individual mouse were incubated with lysed *L. major* promastigote total antigen (ratio: ten parasites per cell), PMA (final concentration 50 ng/ml), or with ConA (final concentration 1 µg/ml) in microtiter plates in RPMI 1640 supplemented with 10% FCS (Sigma), glutamine, Hepes, and Penicillin-Streptomycin (Seromed-Biochrom, Berlin, Germany). After 72 h, cells were harvested and the percentage of proliferating B cells, CD4^+^ and CD8^+^ T cells was determined by flow cytometry. For the analysis of human cytokine production in *L. major* infected humanized mice, spleen cells (three weeks post infection) were harvested and re-stimulated as described above. Supernatants were collected 72 hours later and the release of cytokines was measured in a 5-colour-Multiplex Human Cytokine Panel-assay (Milllipore, Billerica, MA, USA) according to manufacturer's protocol.

### Flow cytometry analysis

Reconstitution with human immune cells was determined by flow cytometry using a LSR-II flow cytometer running the Diva software package (BD Biosciences, San Jose, USA). To reduce non-specific binding, cells were incubated with mouse and human IgGs (10 ug/ml, Sigma) on ice for 10 minutes before staining with human specific monoclonal antibodies (mAb) or appropriate isotype mAb. Samples were stained using the following human specific mAb: anti-CD3-FITC (IgG2aκ, clone HIT3a), anti-CD19-PE (IgG_1_κ, Clone: HIB19), anti-CD33-PerCP-Cy5.5 (IgG_1_κ, clone P67.6), anti-CD45-APC (IgG_1_κ, clone HI30), anti-CD4-PE (IgG_1_κ, clone SK3), and anti-CD8-PE (IgG_1_κ, clone HIT8a) from BD Biosciences. For further characterization we used anti-CD3-PerCP (IgG2a, clone OKT3), anti-HLA-DR-PE-Cy7 (IgG2_b_κ clone LN3), anti-CD86-PE (IgG2_b_κ clone IT2.2), anti-CD45RA-PE-Cy7 (IgG2aκ, clone HI100), and anti-CD27-Biotin (IgG_1_κ, clone O323) from eBioscience (San Diego, USA).

### Immunofluorescence microscopy

Biopsies from organs of interest were embedded in Killik cryostat embedding medium (BioOptica, Milano, Italy) and stored at −80°C. Cryo sections (7 µm) were thawed onto poly-L-Lysin slides (Thermo Scientific, Germany) and processed as described before [38]. The sections were first stained with rabbit-anti-*L. major* serum in PBS/BSA and 0.1% saponin (Roth, Karlsruhe, Germany). After washing in PBS containing 0.01% Tween-20, slides were incubated with goat anti-rabbit AlexaFlour 546 (Invitrogen, Darmstadt, Germany). The antibodies anti-hCD45 (APC) antibody (IgG1κ, clone HI30) and anti-hCD68-PE (IgG2bκ, clone Y1/82A) were used to stain for human leukocytes and macrophages. All antibodies were tested for human-specificity on not humanized NSG mice. Nuclei were stained using DAPI (Sigma-Aldrich, Deisenhofen, Germany). After mounting with Permafluor (Thermo Scientific), sections were analyzed using an immuno-fluorescence microscope (Zeiss, Jena, Germany) equipped with high-sensitivity gray scale digital camera (Openlab System; Improvision, Heidelberg, Germany). Separate images were collected for each section, analyzed and merged false-color afterwards. Final image processing was performed using Adobe Photoshop Elements (Adobe Systems GmbH, München, Germany).

### Liver damage

Alanin-aminotransferase (ALT/GPT), and aspartat-aminotransferase (AST/GOT) were measured in serum using the analytic instrument COBAS INTEGRA 800 (Roche Diagnostics, Germany). Normal values published for mice: ALT = 17–77 (U/I) and AST = 54–298 (U/I) and for humans: ALT = <50 and AST = <50.

### Nitric oxide synthase 2 (iNOS) mRNA expression analysis

Spleen and foot tissue or spleen cells were homogenized in a Tissue Lyser (Qiagen, Hilden, Germany), total RNA was extracted using the TriFast reagent (Peqlab, Erlangen, Germany) and contaminating DNA was removed with DNase I (Ambion DNAfree, Invitrogen, Karlsruhe, Germany). Subsequently, 2–5 µg RNA were reverse transcribed using the High Capacity cDNA Reverse Transcription Kit (Invitrogen). To assess the amount of iNOS cDNA the Applied Biosystems HT7900 Taqman quantitative PCR system (Invitrogen) was used. Human and mouse NOS2 cDNA was measured in triplicates with the following gene-specific Applied Biosystems Taqman assays (Invitrogen): huNOS2 (Hs01075529_m1), mNOS2 (Mm00440485_m1). The gene for human glyceraldehyde 3-phosphate dehydrogenase (GAPDH, Hs02758991_g1) or for mouse hypoxanthine guanine phosphoribosyl transferase-1 (HPRT-1, Mm00446968_m1) was used as endogenous control for calibration of mRNA levels, respectively. Quantitative analyses were performed using the SDS 2.3 software (Applied Biosystems/Invitrogen). The mRNA levels were calculated by the following formula: relative expression = 2-(CT(Target)-CT(Endogenous control))×f, with f = 10^4^ as an arbitrary factor.

### Statistics

Statistical analysis was performed using *Graph Pad's Prism*. All data are represented as as mean ± SEM, and were tested for statistical significance using Student's t test, ANOVA, Bonferroni posttest, Log-rank or Tukey's Multiple Comparison Test , as indicated in the figure legends.

## Results

### Humanized mice infected with *Leishmania major* showed dose-dependent footpad swelling and disease progression

**Figure 1:**
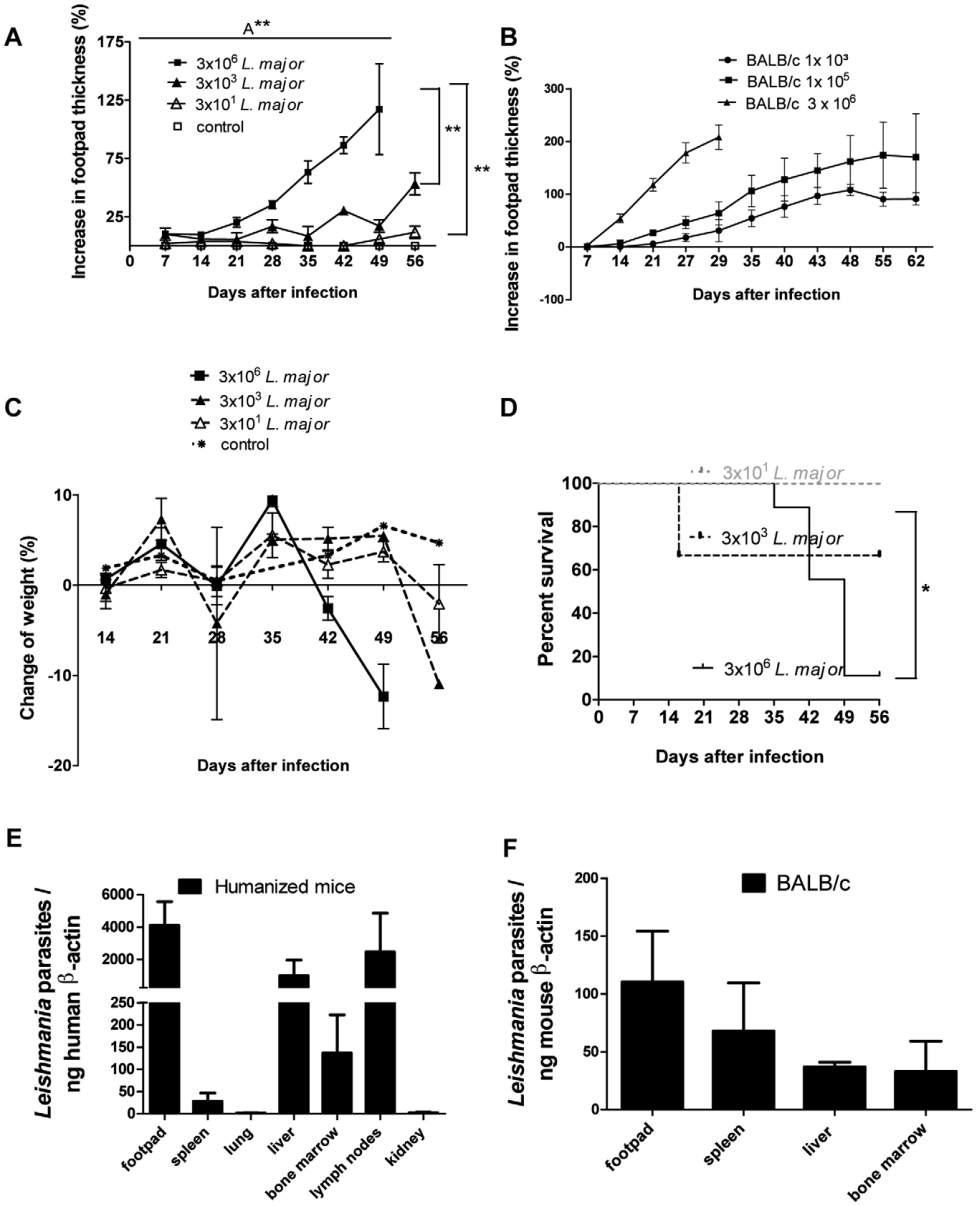
Humanized mice infected with *Leishmania major* showed dose dependent footpad swelling, weight loss, survival, and parasite infestation. Humanized mice (A, C, E) and BALB/c (B, D, F) mice (at the age of 3 months) were infected with different numbers of *L. major* parasites and were weekly monitored for footpad swelling (A; n = 11 and B n = 9), weight changes (C; n = 11), survival (D; n = 15). The amount of *L. major* parasites was normalized to human β-actin in 3×10^6^ *L. major* infected humanized mice (D; n = 7 in spleen, liver, lung and bone marrow; n = 6 in kidney; n = 4 in footpad; n = 3 in lymph node). Three 3×10^6^ *L. major* infected BALB/c mice served as control and parasite load was normalized to mouse β-actin (Figure 1F). Error bars represent means ± SEM (standard error of the mean). Significances between groups were analyzed in one-way Anova (A** = p = 0,0013). Additionally significances between groups are marked with ** (p<0,01) analyzed with Tukey's Multiple Comparison Test. Survival curve was analyzed by Log-rank (Mantel-Cox) Test (* = p<0,05).

Shortly before infection humanized mice were tested for efficient reconstitution with human immune cells (Figure S1). To prove the concept that humanized mice develop a local inflammation at the site of infection we infected them subcutaneously with different numbers of *L. major* parasites. Depending on the amount of parasites injected, differences in footpad swelling (Figure 1A) and weight loss (Figure 1C) were found. Humanized mice infected with a high dose (3×10^6^) of *L. major* showed an earlier, massive weight loss starting at day 35 followed by intermediate dose (3×10^3^) which resulted in a delayed, less pronounced weight loss at day 49 post infection (Figure 1B). Due to the severity of disease, some of the animals infected with 3×10^6^ or 3×10^3^ *Leishmania* parasites died between day 14 and day 49 and the remaining mice were sacrificed at day 50 and 56, respectively (Figure 1D). In contrast to the high dose infection, low dose infection (3×10^1^) caused only a slight weight loss (Figure 1C) and all mice survived until day 56 (Figure 1D). Non-infected humanized mice served as controls and did not show any sign of weight loss or disease and survived until day 56 on which they were sacrificed (data not shown).

In comparison high dose (3×10^6^) infected BALB/c showed the highest local inflammation reaction and had to be sacrificed after day 29 (Figure 1B). Six weeks post infection, quantification of the number of *Leishmania* parasites within the samples of humanized mice revealed that the site of infection represents the organ with the highest parasites load (Figure 1E). Visceral organs such as the spleen and liver were also infected but with lower concentration of parasites when normalized to human β-actin (Figure 1E). High dose infected BALB/c showed also systemic spreading of the parasites with the highest concentration in the footpad (Figure 1F).

Considering the high load of *L. major* parasites in the liver of humanized mice, we assessed the liver damage by measuring the levels of the liver enzymes Aspartat-Aminotransferase (AST/GOT) and Alanin-Aminotransferase (ALT/GPT) in the serum. These data revealed that humanized mice show an increase of GOT (ø 541 U/l+/−220 SEM) and GPT (ø70 U/l+/−19 SEM) after infection with *L. major* compared to uninfected controls GOT (ø 77,2 U/l+/−6 SEM) and GPT (ø 21 U/l+/−2,6 SEM). In contrast, in resistant C57BL/6 mice (GOT: 88 U/l+/−3 SEM; GPT: 15 U/l+/−0,6 SEM) as well as susceptible BALB/c (GOT: 198 U/l+/−47 SEM; GPT: 71 U/l+/−15 SEM) infected with *L. major* parasites no signs of liver damage were detectable. Thus, only humanized mice infected with *L. major* showed an increase in GOT levels compared with normal values of GOT and GPT in humans and mice (see Material & Method section).

Notably, these infection experiments clearly demonstrate that humanized mice show signs of inflammation at the site of infection indicating that they respond to the protozoan parasites *L. major*. Furthermore, we show that comparable to some human patients [39], [40] humanized mice develop a mixture of cutaneous and visceral manifestation of leishmaniasis. The cellular components involved in that process are presented below.

### *Leishmania major* parasite infestation of human cells in the secondary lymphoid organs and at the site of infection

**Figure 2:**
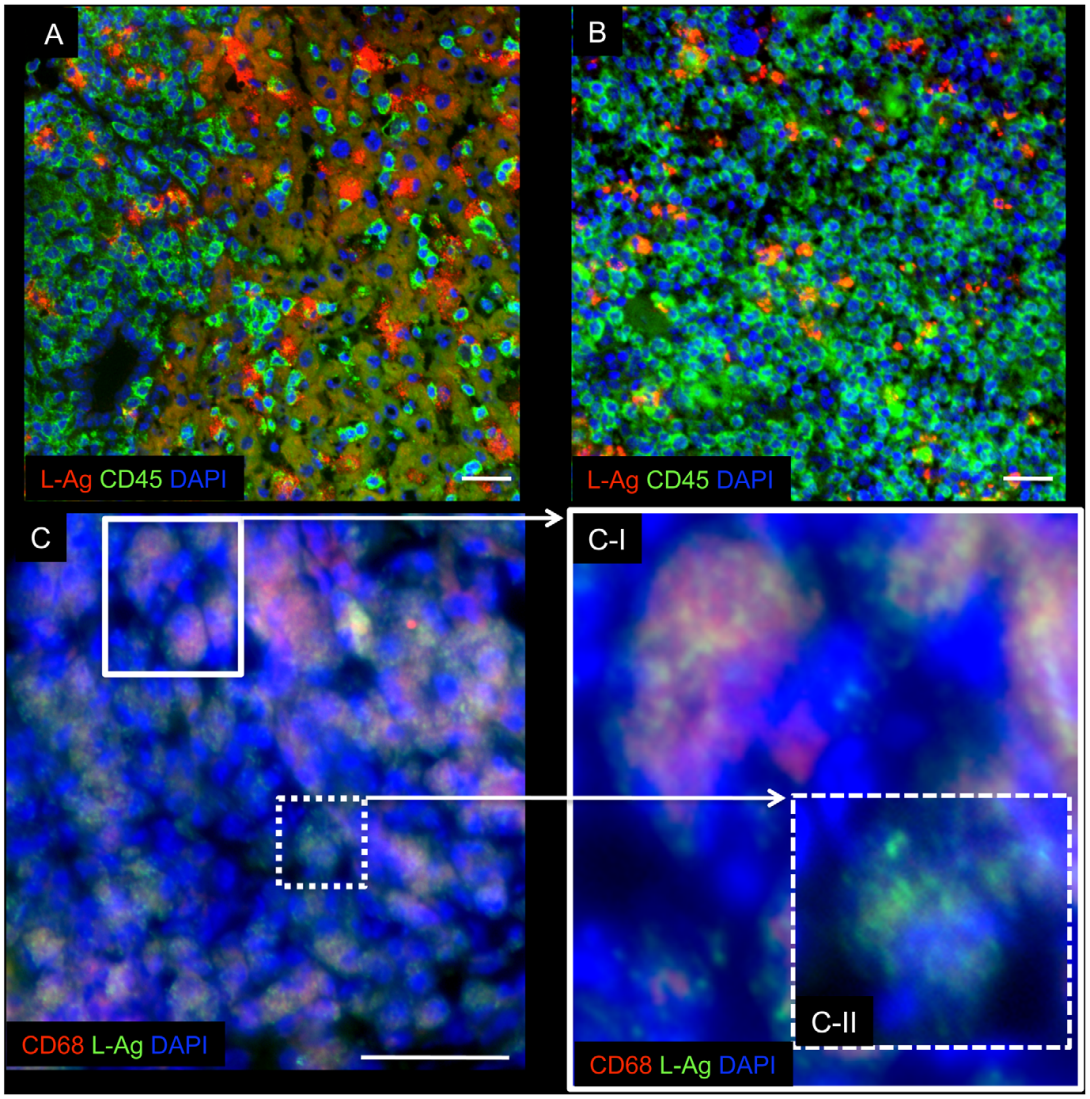
Human-derived immune cells harbour *Leishmania* parasites in humanized mice. 42 days after high dose infection (3×10^6^ *L. major*), liver spleen and the site of infection were analyzed for the host parasite interaction. Cryosections of liver (A), and spleen (B) biopsies were stained for human CD45 (green), and *Leishmania* antigen (L-Ag, red). *L. major*-infected humanized mice appear in orange (CD45^+^ and L-Ag^+^ colocalization; white arrows). (C) Cryosections of footpad samples (site of infection) were stained for human CD68 (red), and L. antigen (L-Ag, green). The insert C-I highlights infected human CD68^+^ macrophages. The insert C-II highlights infected CD68^−^ cells. The blue-fluorescent DAPI nucleic acid stain was added to all staining (blue; nuclei). Bars represent 50 µm.

To address the question whether human host cells are infected with the parasites, immunohistological analyses were performed. Consistent with the PCR data we were able to show that *L. major* antigens were present in liver and spleen of humanized mice (Figure 2A and B). Furthermore, *L. major* antigens were detectable within human CD45^+^ hematopoietic cells (Figure 2A and 2B). However, there are also murine macrophages and granulocytes (hCD45^−^ cells) still present in humanized mice [21]. Therefore, one cannot exclude the possibility that besides human phagocytes, mouse phagocytes were also infected with *L. major* parasites.

To determine the origin of the infected phagocytes in detail, cryosections from infected footpads and from infected visceral organs (e. g. spleen) were characterized. These data revealed that *Leishmania*-antigens were detectable within nucleated cells (Figure 2C). Moreover, we confirmed that *Leishmania*-antigens were detectable in CD68^+^ human macrophages (Figure 2C; insert CI) and CD68^−^ cells (Figure 2C; insert CII). However, randomized spot tests indicated that over 80% of infected cells were human CD68^+^ macrophages (data not shown). Thus we conclude that human macrophages as well as mouse phagocytes can engulf *Leishmania* parasites within the infected tissue of humanized mice.

To address the questions whether other myeloid-derived human cells respond to the *Leishmania*-infection, antigen-presenting cells such as dendritic cells (Lin^−^CD11c^+^HLA-DR^+^) were characterized in infected humanized mice. These data demonstrate that activation markers such as CD86 and HLA-DR were increased in humanized mice infected with *L. major* compared to uninfected controls (Figure S2 A–C). Therefore, we conclude that myeloid cells derived from human stem cells interact and respond to the parasites *in vivo*.

For functional studies of the leishmanicidal capacity induced at the side of infection and in visceral organs such as the spleen, we analyzed the mRNA expression levels of human and murine derived iNOS three weeks after infection. Naive humanized mice showed a baseline expression of murine iNOS within the footpad and spleen tissue which was significantly increased in the footpad after the infection with *L. major* (Figure S2D) whereas no alterations of mouse iNOS mRNA levels were observed in the spleen. Human iNOS mRNA expression was below the detection limit in all samples (Figure S2D). The fact that mouse iNOS is induced upon infection further supports the idea that not only human macrophages but also murine phagocytes get infected and activated in humanized mice.

### *Leishmania major* infection in humanized mice induced a human-derived adaptive immune response at the site of infection

**Figure 3:**
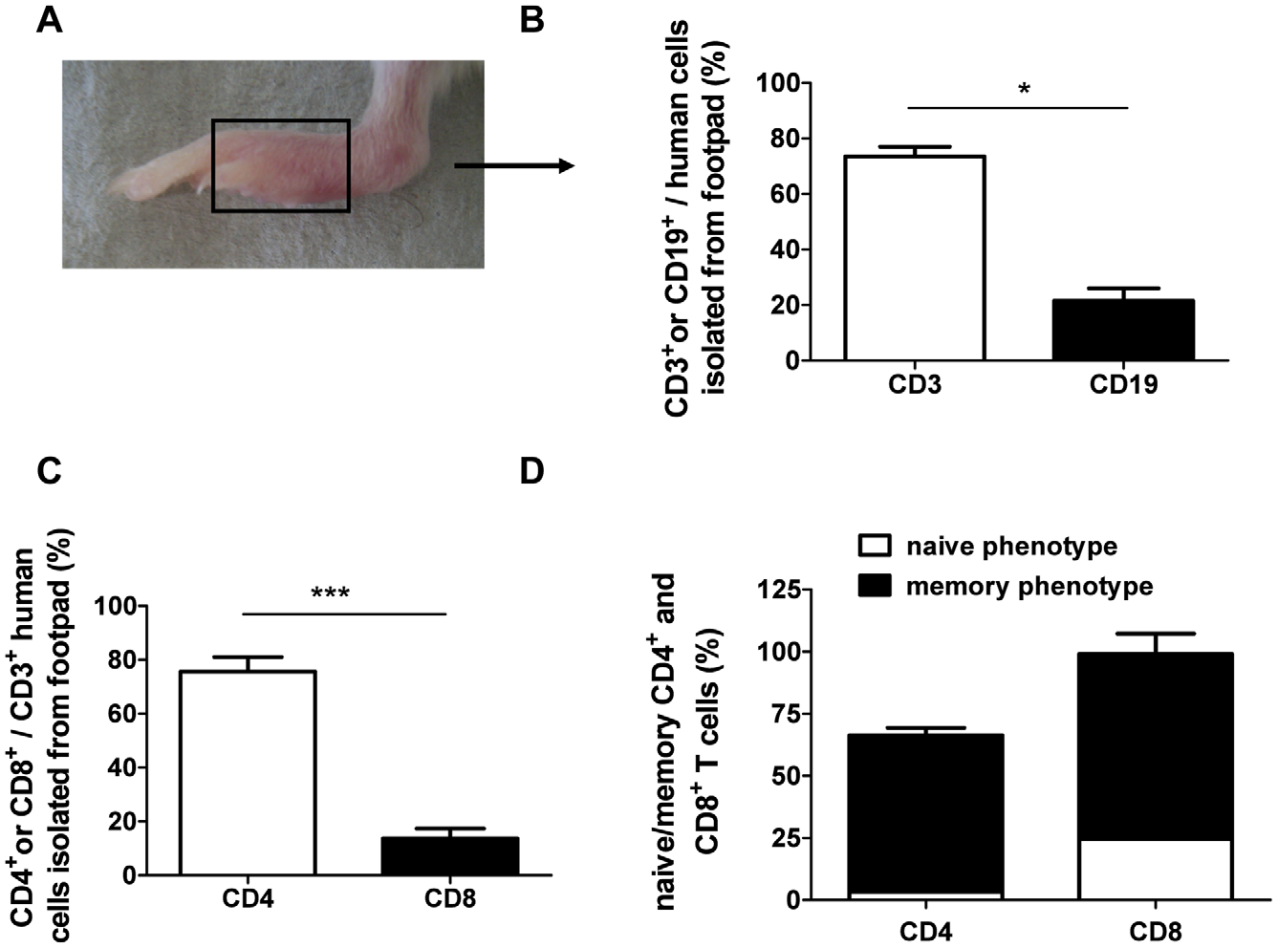
Flow cytometric analyses of human immune cells invading the infected footpad. (A) Infected footpad (3×10^6^ *L. major*) of a humanized mouse is shown. (B) The invasion of human B cells (CD19) and human T cells (CD3) to the site of infection were determined (n = 3). (C) T cells were further characterized for CD4^+^ T helper and CD8^+^ cytotoxic T cell subsets and (D) their naïve (CD27^+^CD45RA^+^) and memory (CD27^+^CD45RA^−^) phenotype. Significances between groups were analyzed using Student's t test (* = p<0.05; ** = p<0.01, n = 3). Error bars represent means ± SEM (standard error of the mean).

*L. major* parasites disseminate within the humanized mice and interact with human leukocytes. We further aimed to answer the question whether the parasites or their corresponding antigens are immunogenic in terms of being able to initiate an adaptive human-derived immune response. From *Leishmania*-infected patients it is known that macrophages represent the dominant cell population within cutaneous lesions, followed by CD3^+^ T cells [41]. Considering our histological findings, demonstrating a dense dermal infiltration of CD68^+^ macrophages building clusters in the infected area (data not shown), we focused our interest on the activation status of human lymphocytes. Comparable to the biopsy data from patients suffering from cutaneous leishmaniasis [41], we were able to show that CD3^+^ T cells dominate the lymphocyte population at the site of infection whereas B cells represent the minority of the skin-infiltrating lymphocytes (Figure 3B). Further distinction of the invading CD3^+^ T cells into CD4^+^ T helper cells versus CD8^+^ cytotoxic T cells revealed the dominance of the CD4^+^ T cell population (Figure 3C). Additional analysis of the infiltrating T cells illustrated a memory phenotype (CD27^+^ CD45RA^−^) of both T cell subpopulations (Figure 3D). This increased population of memory phenotype indicates that T cell activation took place after infection with *L. major* parasites.

### *Leishmania*-specific T cells were generated in humanized mice

**Figure 4:**
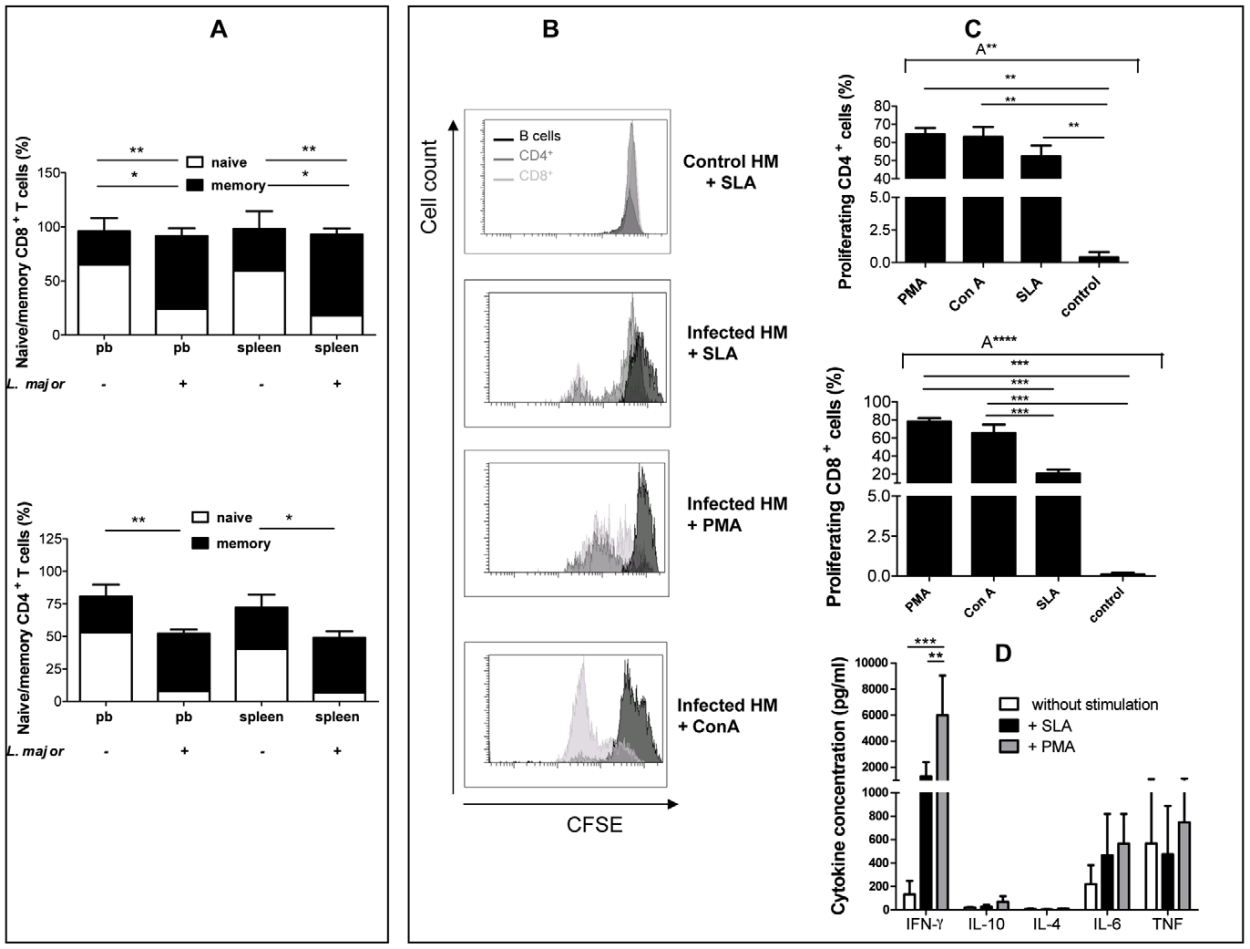
Flow cytometric analyses of human immune cells in the spleen affected by *L. major* infection. (A) Analyses of naïve (CD27^+^CD45RA^+^) and memory (CD27^+^CD45RA^−^) CD8^+^ (top) and CD4^+^ T cells in the spleen with (3×10^6^ *L. major*; n = 9) or without infection (n = 4). (B) CFSE-labelled spleen cells isolated from *L. major*-infected humanized mice (3×10^6^ *L. major*; n = 3) and control humanized mice (HM; without in vivo *L. major* infection; n = 2) re-stimulated with *Leishmania* major antigen (SLA), PMA or ConA for 72 hours. Cell samples were then analyzed for CD4^+^ T cell (light grey), CD8^+^ T cells (grey) and B cells proliferation (dark grey). Percentage of human T cells of three individual humanized mice samples were determined in C. (D) Human cytokine release from spleen cells (isolated from *L. major*-infected (3×10^6^) humanized mice; n = 3) restimulated with soluble *L. major* antigen (SLA), PMA or without restimulation after 72 hours. pb = peripheral blood. Significances between groups were analyzed in one-way (C) or two-way-Anova (A) (A* = p<0,05; A** = p<0,01; A**** = p<0,0001). Additionally significances between groups are marked with * (p<0,05), ** (p<0,01), and *** (p<0,001) analyzed with Tukey's Multiple Comparison Test (1-way-Anova) and Bonferroni posttest (2-way-Anova).

Due to a relatively weak formation of lymph nodes and Peyer's patches in humanized mice [22], we analysed human lymphocytes isolated from spleens of infected animals. The majority of immune cells in the spleen were CD3^+^ T cells, mainly CD4^+^ (70%) in infected humanized mice as well as in controls. Further analysis of CD4^+^ and CD8^+^ T cells revealed that there was a significant switch towards the memory phenotype (CD45RA^−^CD27^+^; Figure 4A). As shown in Figure 4B, immune cells isolated from uninfected controls did not proliferate after stimulation with soluble *Leishmania* antigen (SLA) whereas spleen cells derived from infected animals showed proliferation of CD4^+^ as well as CD8^+^ T cells. The highest proliferation rate was induced by polyclonal stimulation with phorbol myristate acetate (PMA) and Concanavalin A (ConA) (Figure 4B+C). Thus, human T cells are still responsive to different stimuli even though they were embedded in a murine environment.

Furthermore, the cytokine response of splenocytes from infected humanized mice was characterized. These data revealed that a restimulation with SLA results in a significantly increased IFN-γ (Th1 type cytokine) production whereas IL-4 and IL-10 (Th2 type cytokine) were hardly detectable (Figure 4D). The proinflamatory cytokines TNF and IL-6 are not increased after restimulation with SLA. Based on this cytokine profile we conclude that the splenocytes release cytokines representing a Th1-type immune response. In addition we tried to induce a DTH reaction in humanized mice infected with *L. major* to measure the migration capacity of Th1 type effector cell to the site of antigen inoculation [42], [43]. Remarkably, no clinical signs of swelling and redness could be measured after subcutaneous injection of *Leishmania* antigen (data not shown).

### *Leishmania major* load in visceral organs can be reduced by treatment with Miltefosine

Cutaneous leishmaniasis caused by *L. major* is characterized by skin ulcers, papules or nodules. However, exceptions from these classical cutaneous manifestations have been reported. Those patients show unusual forms of cutaneous leishmaniasis with abnormal liver function [39], [44].

As already shown above humanized mice develop a sever course of disease not sufficient to eliminate the parasites. Thus, humanized mice might represent an experimental model for *Leishmania*-caused disease, which do not show any self-healing capacity. Accordingly, we tested a drug-based treatment against the parasites in the humanized mouse model. We used Miltefosine because it is already established for treatment of visceral and cutaneous leishmaniasis [34].

**Figure 5:**
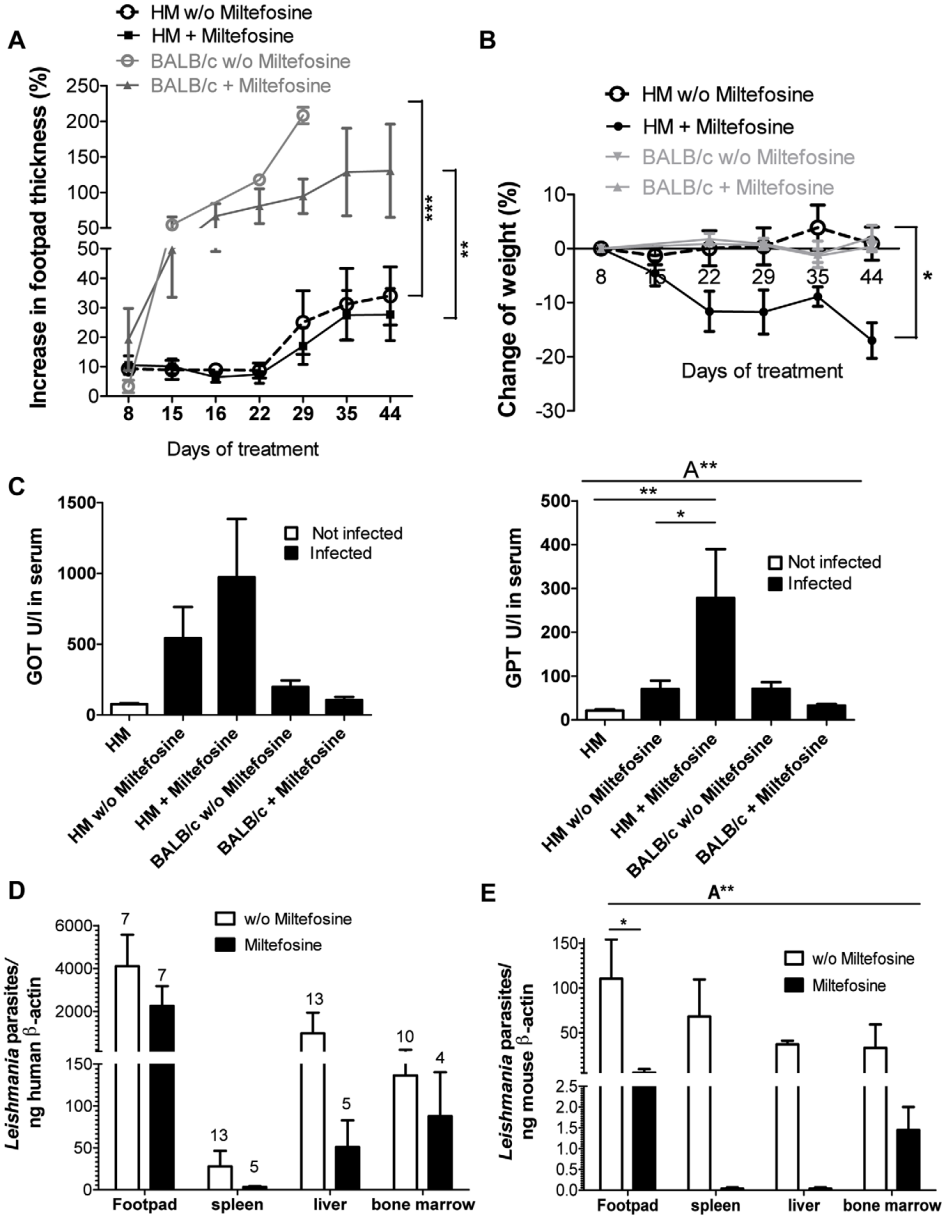
Influences of Miltefosine treatment in infected humanized mice. Footpad swelling (A), weight change (B), and parasitic infestation in different organs normalized to human β-actin (D) in *L. major* (3×10^6^ *L. major*) infected humanized mice with (n = 6) and without Miltefosine (n = 6) treatment. BALB/c mice served as control (n = 3/group). (C) Alanine aminotransferase (ALT/GPT) and aspartate aminotransferase (*AST*/*GOT*) activities in the serum were analyzed in non-infected humanized mice (HM; n = 7), humanized mice infected with *L .major* (3×10^6^ *L. major*) and treated with Miltefosine (HM+Miltefosine; n = 6) and without (HM w/o Miltefosine; n = 14) and compared with BALB/c mice infected without Miltefosine (BALB/c w/o Miltefosine; n = 3) and treated with Miltefosine (BALB/c+Miltefosine; n = 3). (D+E) All mice (BALB/c: n = 3/each group; humanized mice: numbers are indicated above the columns for each organ) were sacrificed six weeks post infection before disease progression led to death of any of the animal. For statistical analyses in A and B, area under the curve was calculated and analyzed using Student's t test (* = P<0.05). Error bars represent means ± SEM (standard error of the mean). Significances between groups were analyzed in one-way (C) and 2-way (D) Anova (A* = p<0,05). Additionally significances between groups are marked with * (p<0,05) analyzed with Tukey's Comparison Test.

To mimic the clinical situation, Miltefosine was given orally starting at day 14 post infection when clinical signs such as swelling and redness at the site of infection were detectable. Miltefosine treatment of humanized and BALB/c mice did not reduce the local inflammatory process at the site of infection (Figure 5A). Nevertheless, humanized mice showed a reduced local inflammation at the site of infection compared to BALB/c. Furthermore, BALB/c mice did not loose weight (with or without treatment) whereas humanized mice lost weight when treated with Miltefosine (Figure 5B). Additionally, only humanized mice treated with Miltefosine revealed increased liver enzyme levels (Figure 5 C).

For the characterization of the therapy efficiency we quantified the number of parasites in the organs of interest. These data revealed that BALB/c mice show significant reduction of the parasite load at the site of infection and visceral organs and bone marrow (Figure 5E). In contrast the Miltefosine therapy in humanized mice showed only slight reduction of the parasites in the liver and footpad (Figure 5D). However, during Miltefosine therapy we observed adverse effects, which are also described in humans [34] such as weight loss (Figure 5B) and significantly increased release of liver enzymes (GOT and GPT), which did not occur in infected and treated BALB/c mice (Figure 5C).

Notably, Miltefosine treatment neither affected the overall reconstitution, the T and B cell distribution nor the priming of human T cells in the spleen (Figure S3A+B).

## Discussion

Humanized mice in triple deficient NOD/*scid*-IL2γ^−/−^ (NSG) mice develop all cellular components of the human immune system such as T, B, and NK cells as well as myeloid cells and are able to mount an adaptive and innate immune response to antigens [19], [21]. Based on this innovative concept to create a human immune system in a murine environment, scientific issues regarding virus-caused infectious diseases [24], [45]–[47], therapy and vaccine studies [28]–[33], graft versus host disease (GvHD) [48], and tumor diseases [37] were already studied.

To characterize the interactions between human immune cells and obligatory intracellular protozoan parasites under *in vivo* conditions we infected humanized mice with *L. major* parasites. In general these parasites induce cutaneous leishmaniasis. However, exceptions of this classic form with mild visceral tendency have been reported in patients before [40], [49]–[51]. This capacity for visceralization was also described in *L. tropica* infected patients [52].

The *L. major* strain (MHOM/IL/81/FE/BNI] used in our experiments was isolated from a LCL patient [53]. Of note, under experimental conditions this *L. major* strain can result in visceral manifestations in resistant C57BL/6 and susceptible BALB/c mice as well [50], [54]–[60]. Thus, it cannot be generally excluded hat *L. major* parasites might show the tendency of mild visceralization in parallel to the cutaneous manifestation.

Here we questioned for the first time whether *L. major* parasites can replicate within humanized mice. Furthermore, we tested this novel experimental model - sharing murine and human components – for its capacity to characterize human adaptive and innate immune response to *L. major* and the efficiency of treatments like such as hexadecylphosphocholine Miltefosine.

Initial experiments revealed that cutaneous inoculation of *L. major* parasites resulted in an inflammation at the site of infection. Additionally the severity of disease correlates with the dose of parasites injected. Thus, humanized mice develop a local inflammation caused by the protozoan parasites. Based on the reconstitution of NSG mice with naïve human leucocytes it is most likely that human cells are involved in the parasite-induced inflammatory response. Thus, we further investigated the phenotype and origin of potential host cells at the site of infection of humanized mice. It is important to mention that humanized mice still possess mouse myeloid cells such as macrophages and granulocytes. And indeed, our data revealed that *Leishmania*-antigens can be detected in human as well as murine myeloid cells indicating that mouse myeloid cells represent target cells for *Leishmania* in humanized mice. As suggested by several findings infected mouse myeloid cells might function as a niche for *Leishmania* allowing immune evasion. First, the human adaptive immune system is not able to control or stimulate mouse immune cells due to mismatched MHC expression and the species-specificity of human IFN-γ [61]–[64]. Second, most of those myeloid mouse cells show functional weaknesses as for instance insufficient differentiation of fully functional DC from NSG bone marrow cells [21]. And third, it has been shown that in NOD-*scid* mice the remaining mouse macrophages show an impaired functionality [61].

However, we detected a 10-fold increase in the expression of murine iNOS mRNA in the footpad tissue of humanized mice three weeks after infection with *L. major* whereas no increase of iNOS mRNA was found in the spleen. This clearly indicates that mouse macrophages somehow get activated during infection, although the upregulation of iNOS mRNA in humanized mice is ∼factor 100 lower than that seen in infected BALB/c mice. In accordance, no iNOS mRNA increase was visible in the spleen of BALB/c mice upon infection [unpublished data, U. Schleicher and C. Bogdan, Erlangen). With regard to parasite control this lack of iNOS increase in the spleen is of minor importance as rather NADPH-oxidase-dependent than iNOS-dependent mechanism are required for the resolution of the parasites in this organ [65]. In contrast to mouse iNOS mRNA no induction of human iNOS mRNA was detectable in infected humanized mice. Since we demonstrated that human macrophages get infected in humanized mice this result rather supports the idea that iNOS expression by macrophages or other cells in response to infections does not occur in the human immune system [15]. That this statement might not hold true in human cutaneous leishmaniasis is supported by results demonstrating iNOS mRNA and protein expression in skin lesions of CL patients [11], [66]. Together, it can be therefore speculated that the atypical visceralisation of *L. major* parasites within humanized mice might be at least partially due to the insufficient production of murine derived leishmanicidal molecules and the lack of human iNOS induction.

Considering that infected macrophages need to be activated by an efficient Th1-cell response [67] the human-derived T cell response was further analyzed. Inflammatory human T cells could be detected at the site of infection. Most of them represent the phenotype of a memory T cells and the majority of these cells belong to the CD4^+^ T helper cell population, known to play a major role in the activation of infected macrophages and subsequent killing of the intracellular *Leishmania* parasites [68]. The fact that CD4^+^ as well as CD8^+^ T cells were efficiently primed *in vivo* indicates that human antigen presenting cells are functional and that the induced human T cell response is *Leishmania*-specific. Based on the fact that splenocytes of infected humanized mice respond with a pronounced human IFN-γ production after restimulation with soluble *Leishmania* antigen (SLA) we conclude that a human Th1-like immune response was induced.

From the experimental model of leishmaniasis it is known that resistant C57BL/6 mice develop a delayed hypersensitivity (DTH] response after s.c. injection of SLA whereas susceptible BALB/c mice do not show any signs of a DTH response [42], [43]. This lack of DTH reaction was also detectable in infected humanized mice and might represent the dysfunction of the induced Th1-type effector cells to get recruited to the site of antigen inoculation.

Based on the fact that humanized mice do not show signs of a DTH reaction we can conclude that Th1 cells are efficiently primed within the spleen but failed to migrate to the site of the infection. This might also explain the relative low parasite density within the spleen compared to the parasite load detectable at the site of infection.

To investigate the versatility of our model for future drug studies, we treated infected humanized mice and BALB/c mice with Miltefosine. In BALB/c mice the application of Miltefosine induced a reduction of the parasite load predominantly in visceral organs such as liver and spleen in the absence of liver damage and weight loss. In contrast humanized mice respond different to the Miltefosine therapy. They show signs of liver damage and significant weight loss after treamtent. Thus the Miltefosine-derived side effect must be the consequence of the presence of human immune cells. This is in agreement with published data [69]–[70] demonstrating signs of liver toxicity in patients after Miltefosine therapy. The observed Miltefosine-based side in humanized nice might be due to the fact that Miltefosine can activate human immune cells to release pro inflammatory cytokines [71]–[72] that in turn cause the observed liver damage. Thus, humanized might be a useful tool to test possible side effects which are not detectable in classical animal models such as BALB/c, before starting clinical trials.

The leishmanicidal effect of Miltefosine in humanized mice is visible but not comparable to control BALB/c mice. This might be due to the lack of mouse IFN-γ which in turn can not activate infected mouse macrophages.

In conclusion, we have generated a novel *Leishmania major* infection model using humanized mice which develop a systemic non-self-healing disease. Humanized mice might therefore be especially interesting for studies on visceral leishmaniases offering an additional challenge for new treatment strategies: the elimination of resting (“hiding”) *Leishmania* parasites within the mouse immune cells. In addition, side effects induced by activated human immune cells (cytokine release) which do not occur in wild type mice can now be illuminated in a small animal model which might help to predict possible side effects before the drugs get tested in clinical trials.

## Supporting Information

Figure S1 **Transplantation of human CD34^+^ into neonatal NSG mice resulted in stable human engraftment throughout all organs.** (A) Stable reconstitution of human CD45^+^ cells was detectable in the peripheral blood in humanized mice starting with the age of six weeks up to more than sixteen weeks. (B) In humanized mice, transplantation of 3×10^5^ human stem cells induces human engraftment (CD45^+^) and distribution in all organs in the age between eight to sixteen weeks. (C) Further immunohistological staining in different organs (here shown for spleen) stained clusters of human hematopoietic cells (CD45), human B cells (CD79A), human macrophages (CD68), and human T cells (CD3).
(TIF)

Figure S2 ***Leishmania major*** **infection in humanized mice induced human innate immune response.** (A, B) Characterization of human dendritic cells (Lin^−^ CD11c^+^ HLA-DR^+^) and (C) its activation status (CD86 and HLA-DR expression) in the spleen with (+) and without (−) *L. major* infection. (D) Relative mouse and human iNOS expression in spleen and footpad (skin) of 3 weeks infected humanized mice were analyzed by quantitative PCR. Error bars represent means ± SEM (standard error of the mean). Error bars represent means ± SEM (standard error of the mean). Significances between groups were analyzed in 1-way (B) and 2-way Anova (C). Significances between groups (n = 3) are marked with * (p<0,05) and ** (p<0,01) analyzed with Bonferroni posttest.
(TIF)

Figure S3 **Miltefosine treatment did not have significant influence on reconstitution level and immune cell distribution in the spleen.** Flow cytometric analyses of spleen cells isolated from *L. major* infected humanized mice treated with Miltefosine in comparison with not treated humanized mice (Aqua). (A) Spleen cells were analyzed for total reconstitution level (CD45^+^), T cells (CD3^+^) and B cells (CD19^+^). (B) T cells were further characterized for CD4^+^ and CD8^+^ subsets and naïve (CD27^+^CD45RA^+^) and memory (CD27^+^CD45RA^−^) phenotype. Error bars represent means ± SEM (humanized mice+Miltefosine; n = 6 and humanized mice+Aqua; n = 6).
(TIF)
