# Supplementary figures and images for: Leishmania major Infection in Humanized Mice Induces Systemic Infection and Provokes a Nonprotective Human Immune Response
Source: PLoS Negl Trop Dis. 2012 Jul 24;6(7):e1741. doi: 10.1371/journal.pntd.0001741 (PMC3404120; doi:10.1371/journal.pntd.0001741)

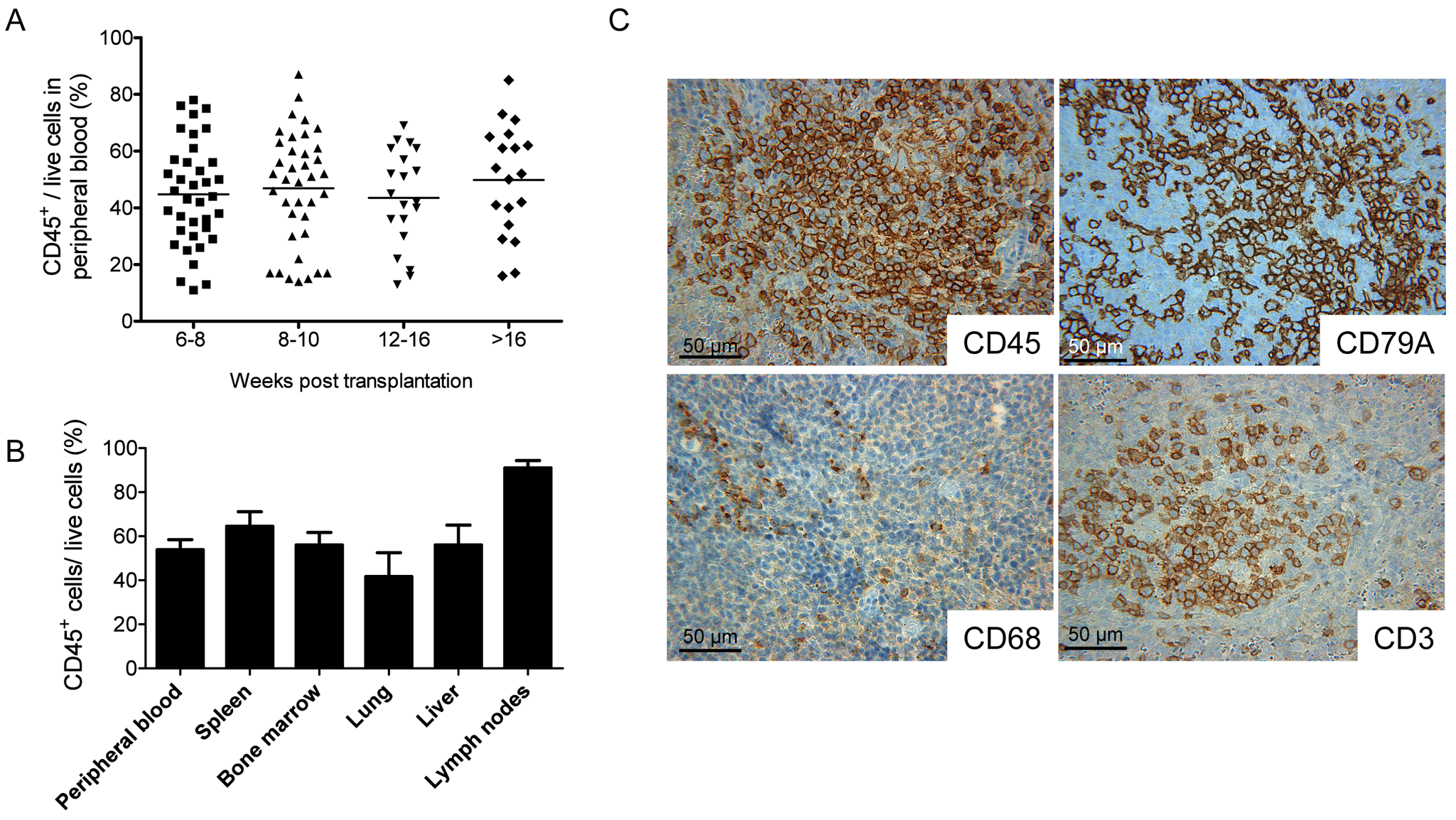

Supplement: Figure S1 — Transplantation of human CD34+ into neonatal NSG mice resulted in stable human engraftment throughout all organs. (A) Stable reconstitution of human CD45+ cells was detectable in the peripheral blood in humanized mice starting with the age of six weeks up to more than sixteen weeks. (B) In humanized mice, transplantation of 3×10^5^ human stem cells induces human engraftment (CD45+) and distribution in all organs in the age between eight to sixteen weeks. (C) Further immunohistological staining in different organs (here shown for spleen) stained clusters of human hematopoietic cells (CD45), human B cells (CD79A), human macrophages (CD68), and human T cells (CD3). (TIF) [file pntd.0001741.s001.tif]

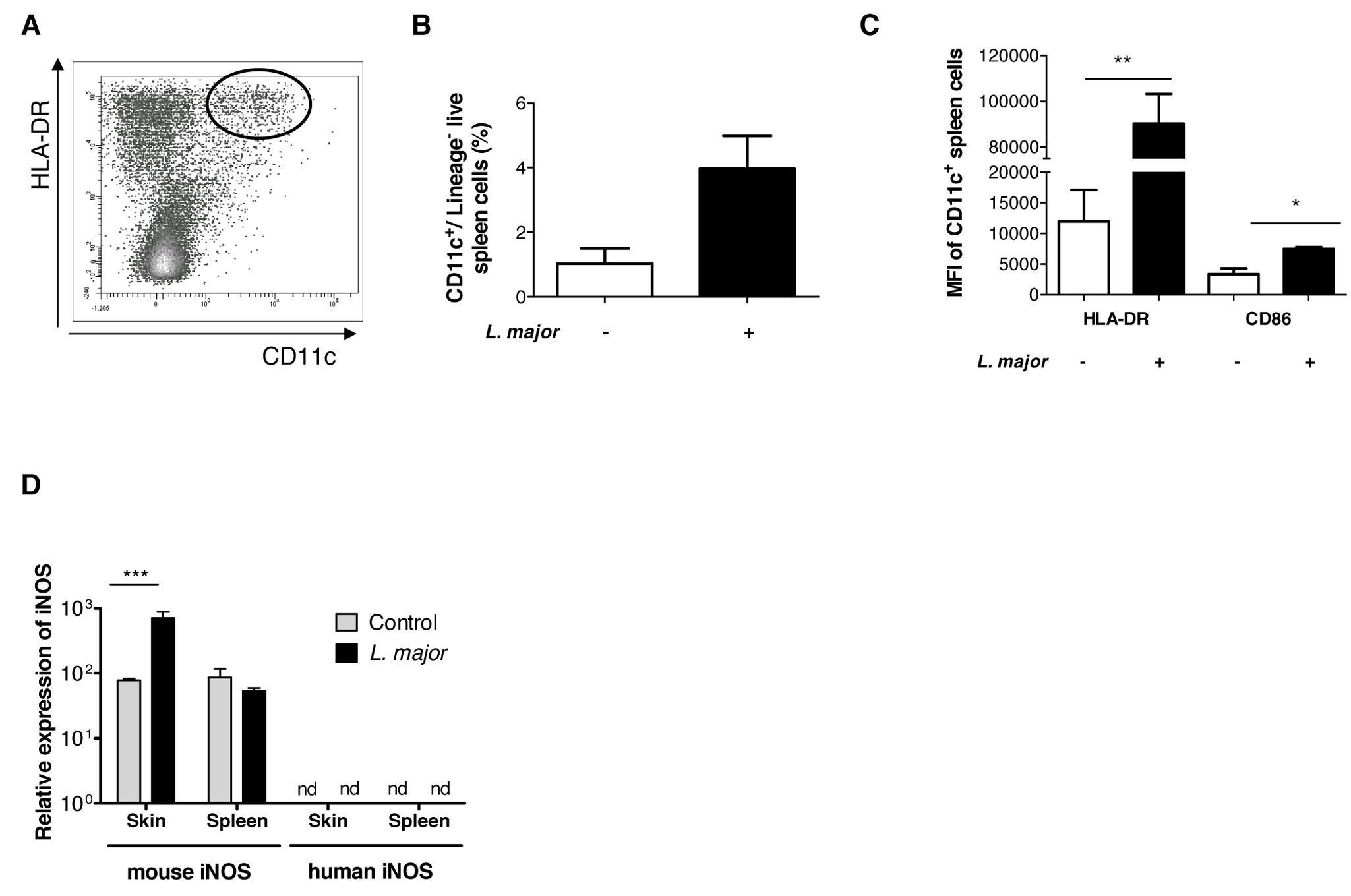

Supplement: Figure S2 — Leishmania major infection in humanized mice induced human innate immune response. (A, B) Characterization of human dendritic cells (Lin− CD11c+ HLA-DR+) and (C) its activation status (CD86 and HLA-DR expression) in the spleen with (+) and without (−) L. major infection. (D) Relative mouse and human iNOS expression in spleen and footpad (skin) of 3 weeks infected humanized mice were analyzed by quantitative PCR. Error bars represent means ± SEM (standard error of the mean). Error bars represent means ± SEM (standard error of the mean). Significances between groups were analyzed in 1-way (B) and 2-way Anova (C). Significances between groups (n = 3) are marked with * (p<0,05) and ** (p<0,01) analyzed with Bonferroni posttest. (TIF) [file pntd.0001741.s002.tif]

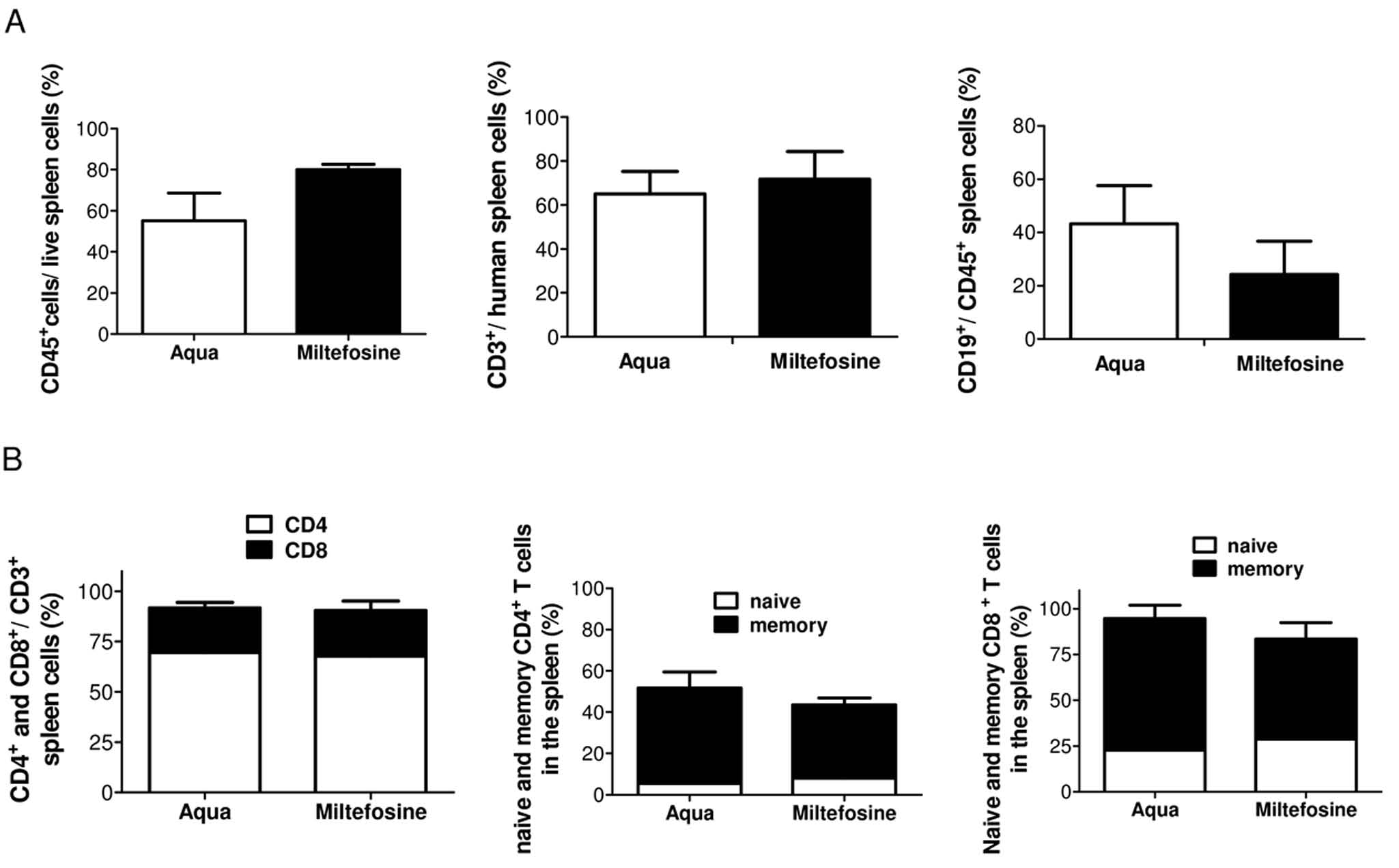

Supplement: Figure S3 — Miltefosine treatment did not have significant influence on reconstitution level and immune cell distribution in the spleen. Flow cytometric analyses of spleen cells isolated from L. major infected humanized mice treated with Miltefosine in comparison with not treated humanized mice (Aqua). (A) Spleen cells were analyzed for total reconstitution level (CD45+), T cells (CD3+) and B cells (CD19+). (B) T cells were further characterized for CD4+ and CD8+ subsets and naïve (CD27+CD45RA+) and memory (CD27+CD45RA−) phenotype. Error bars represent means ± SEM (humanized mice+Miltefosine; n = 6 and humanized mice+Aqua; n = 6). (TIF) [file pntd.0001741.s003.tif]
